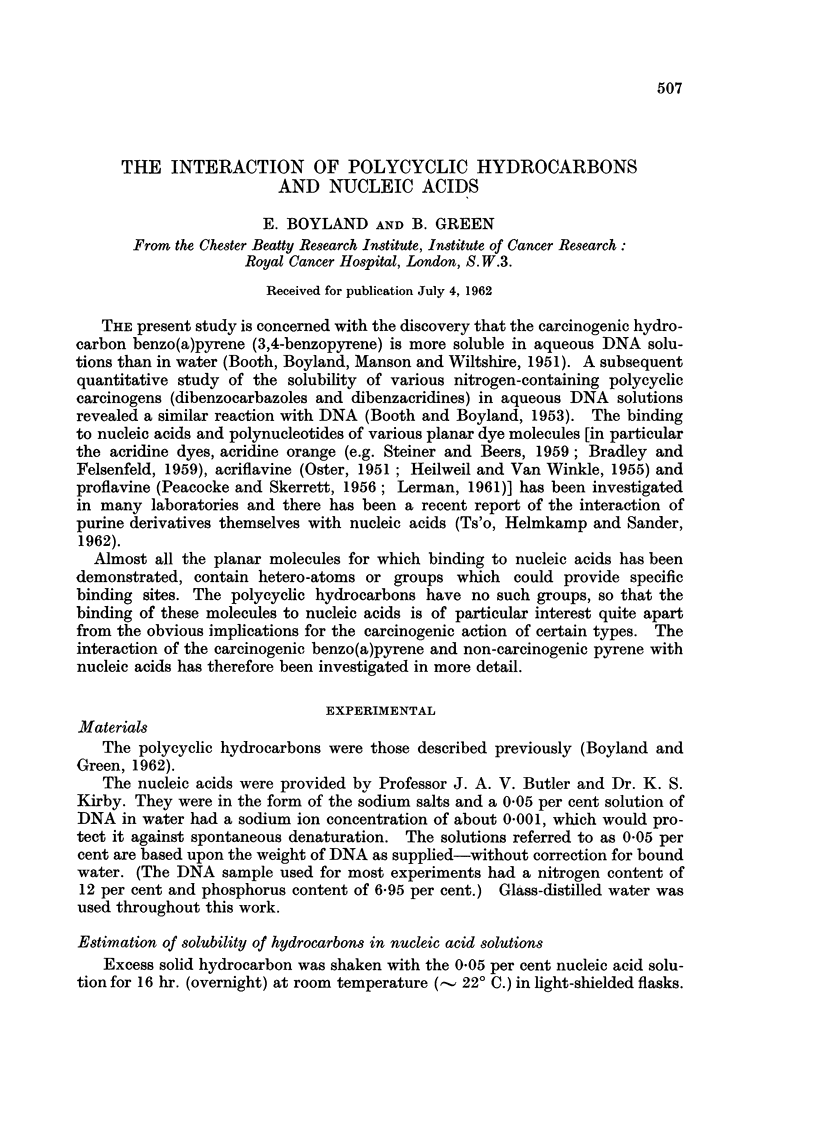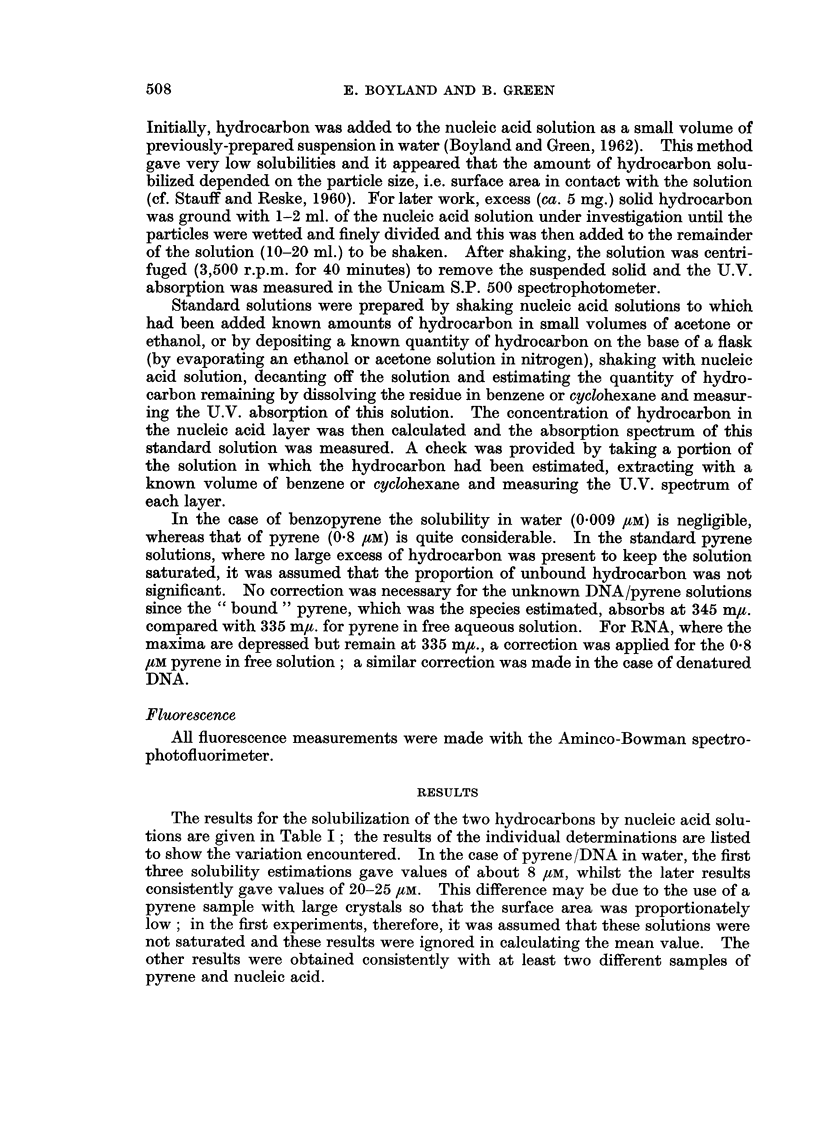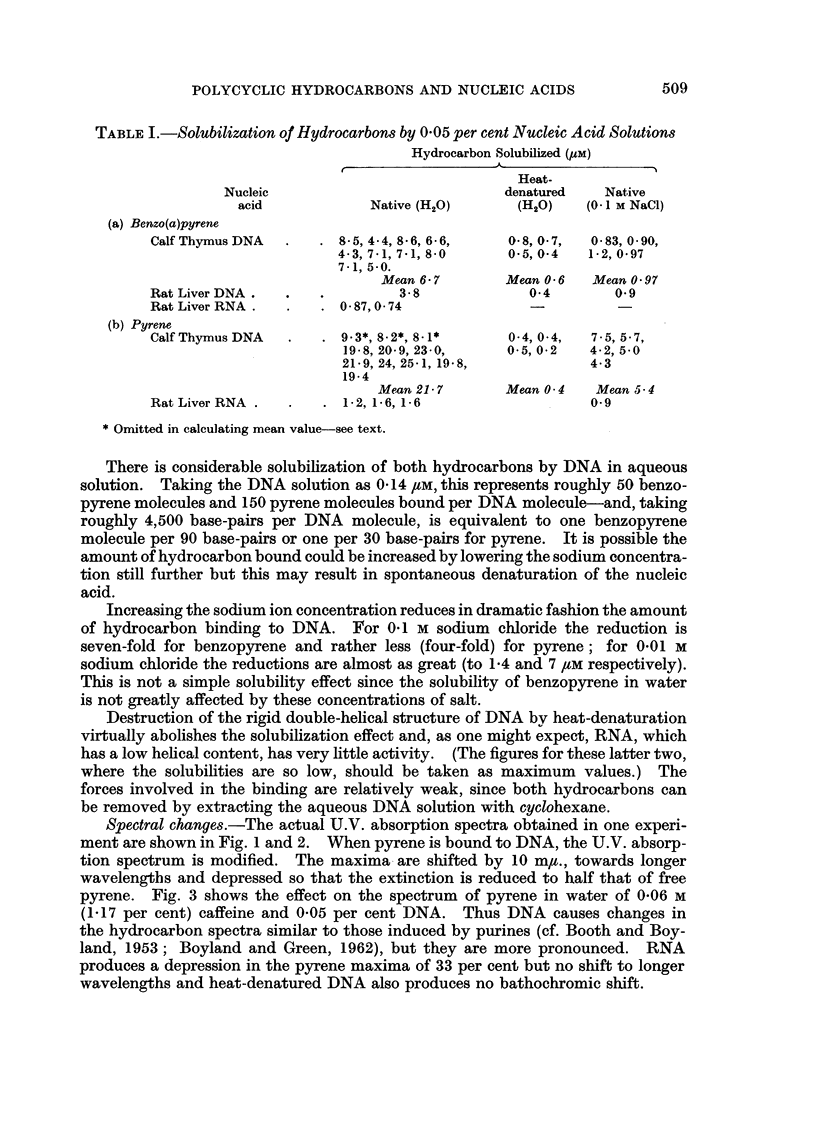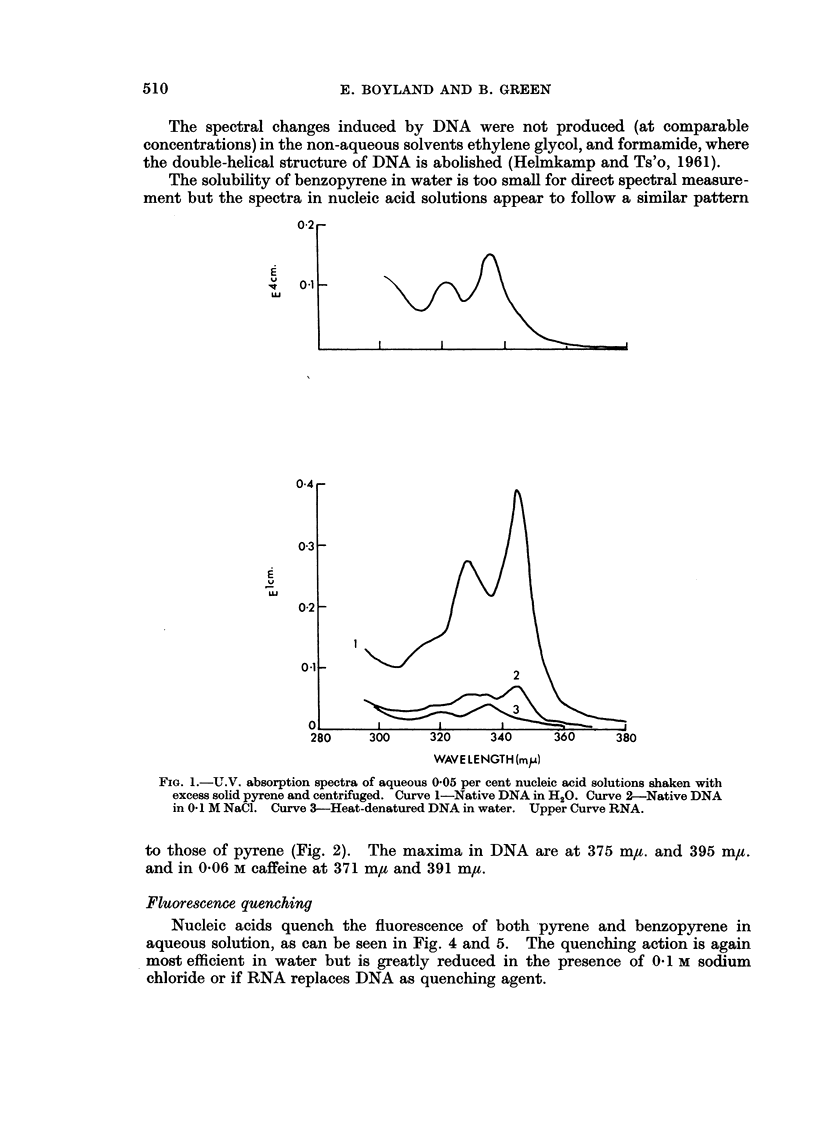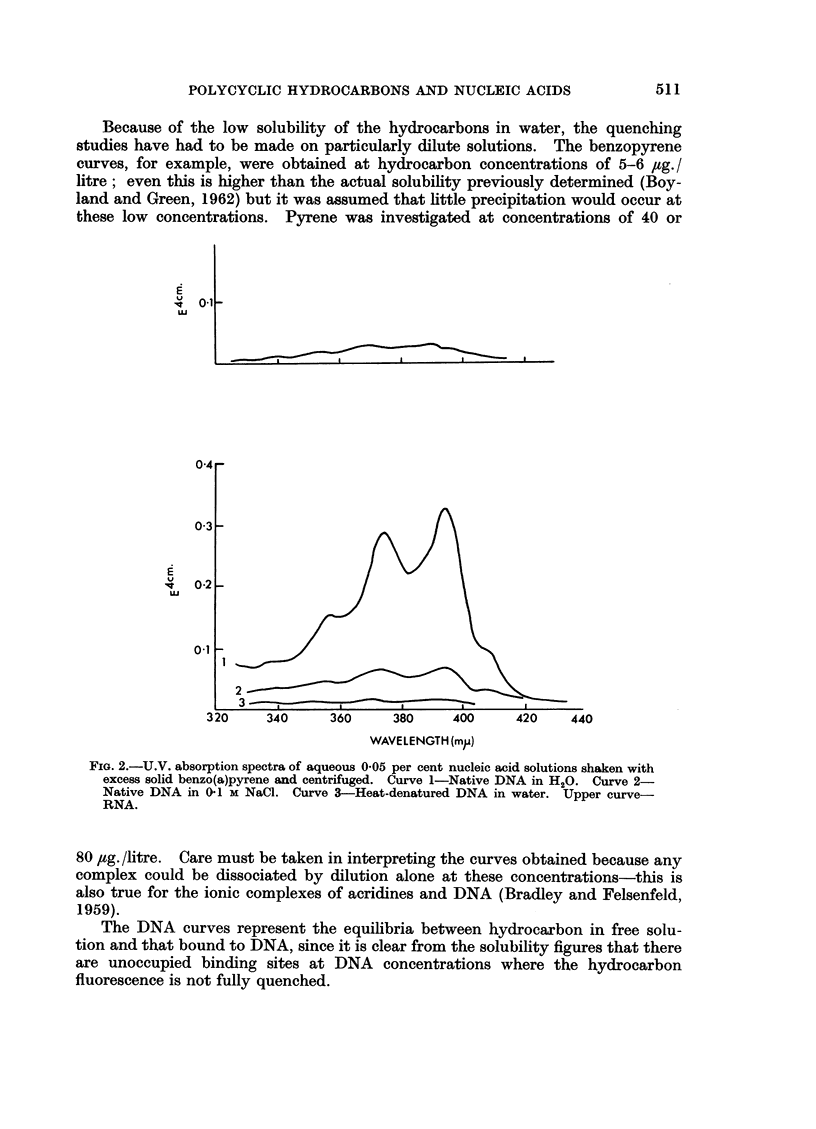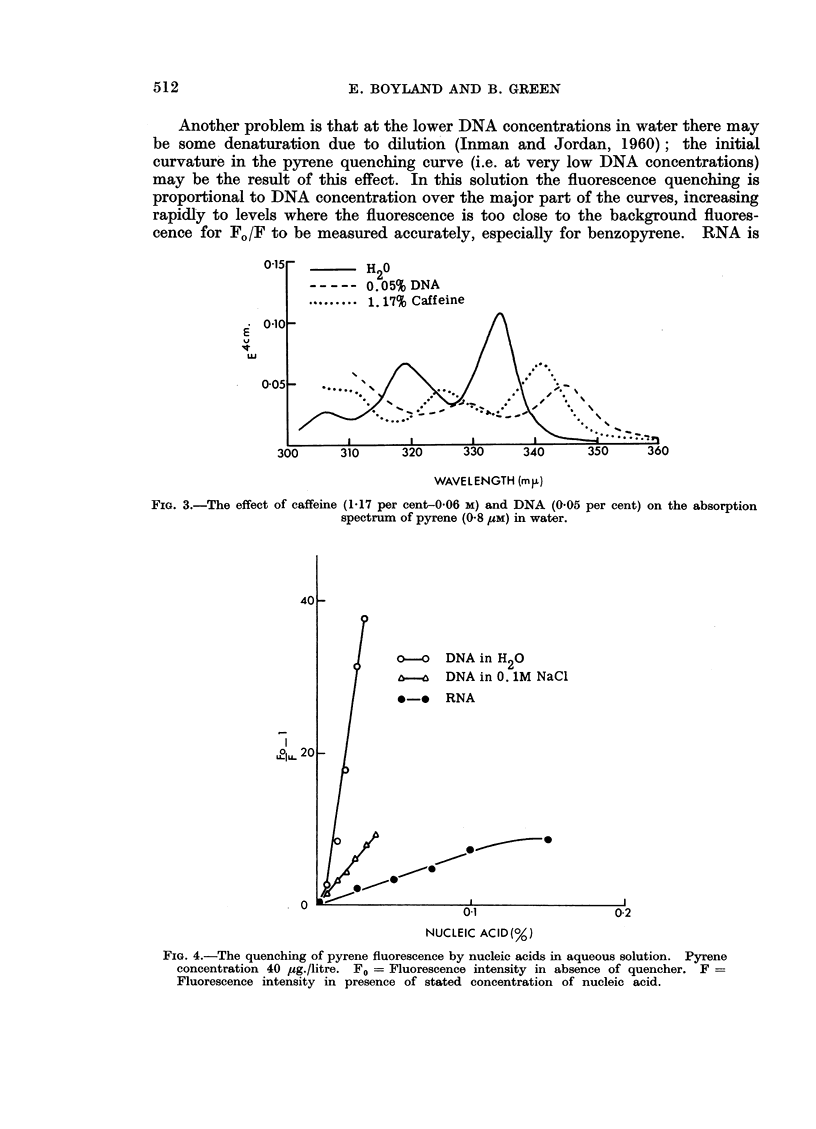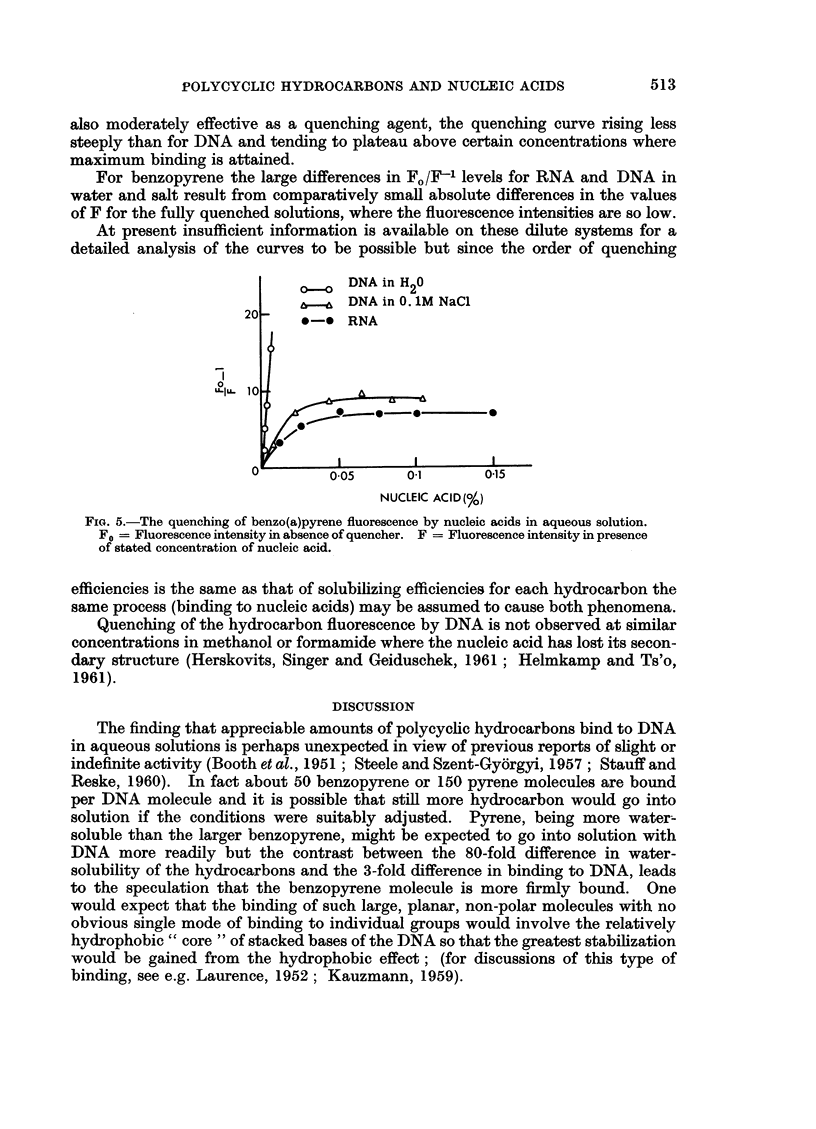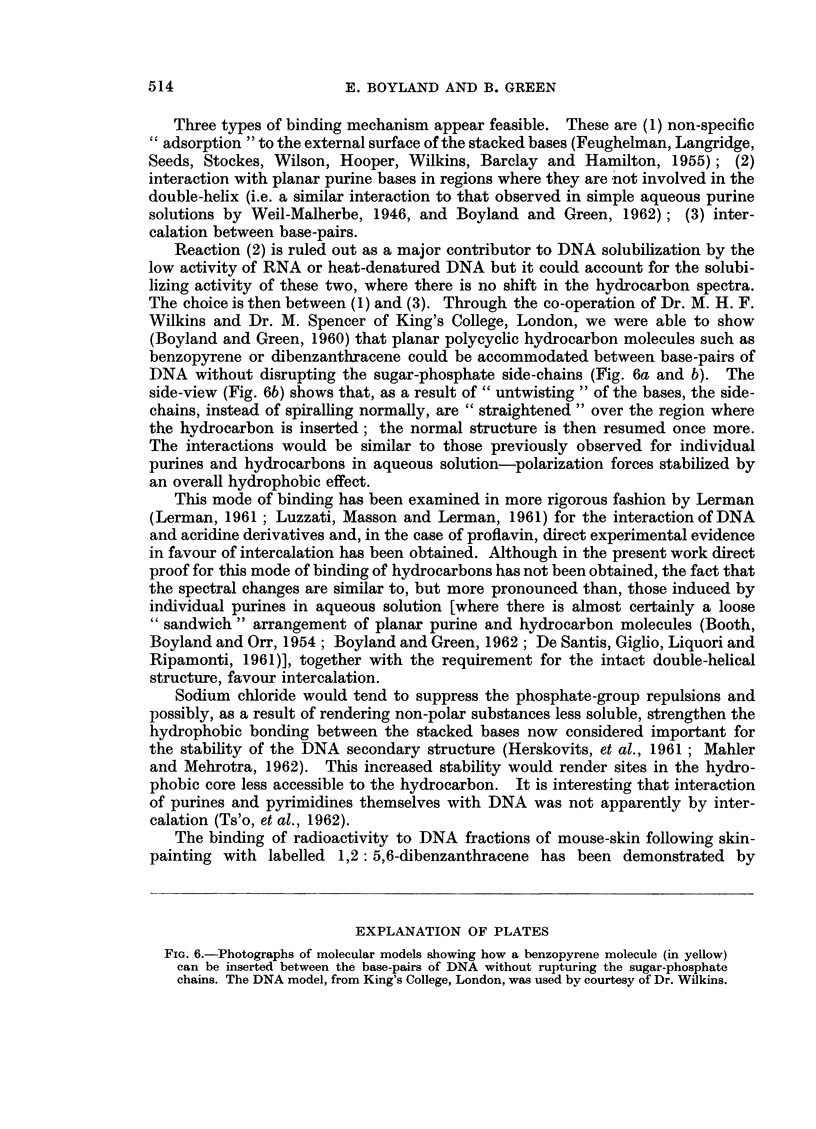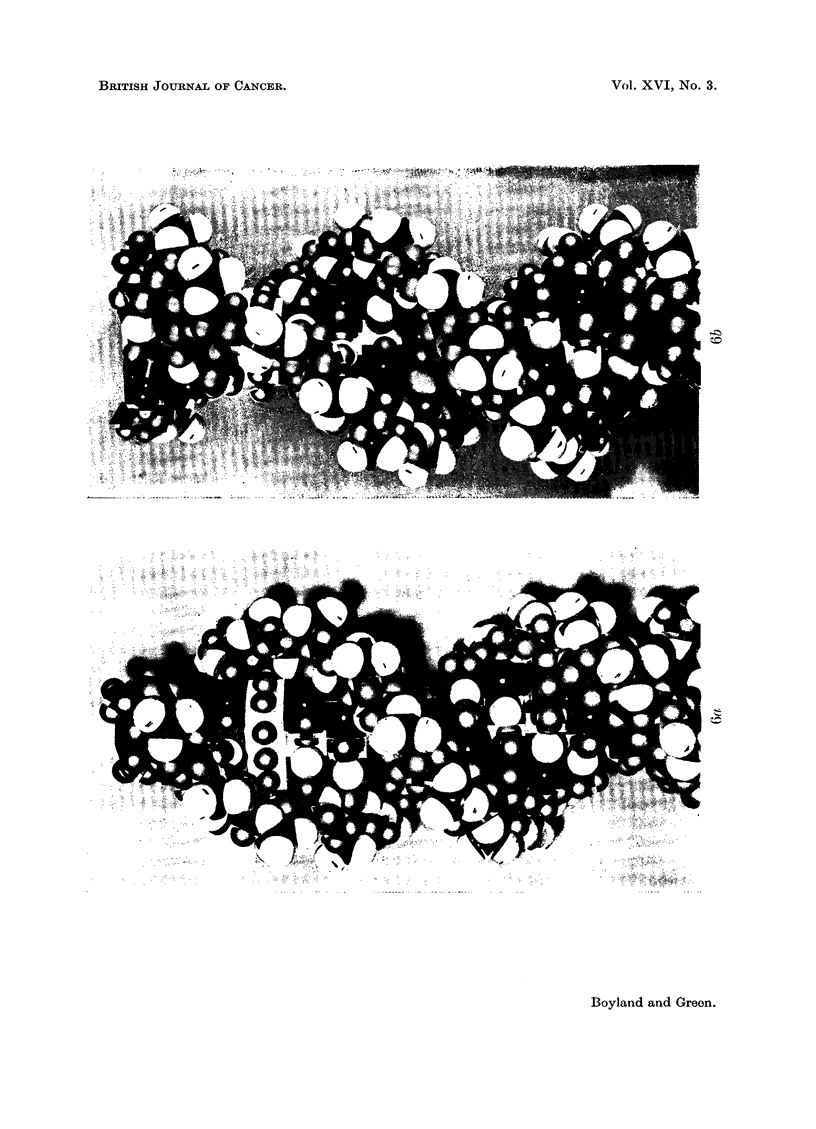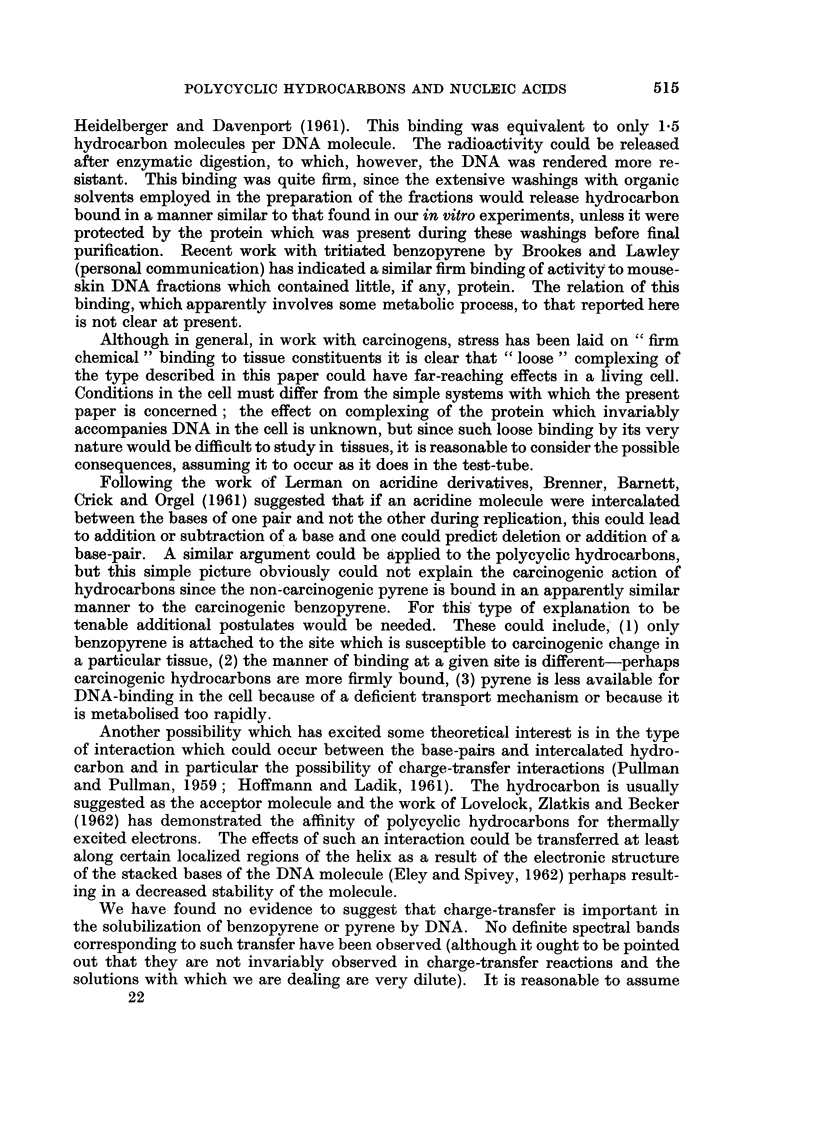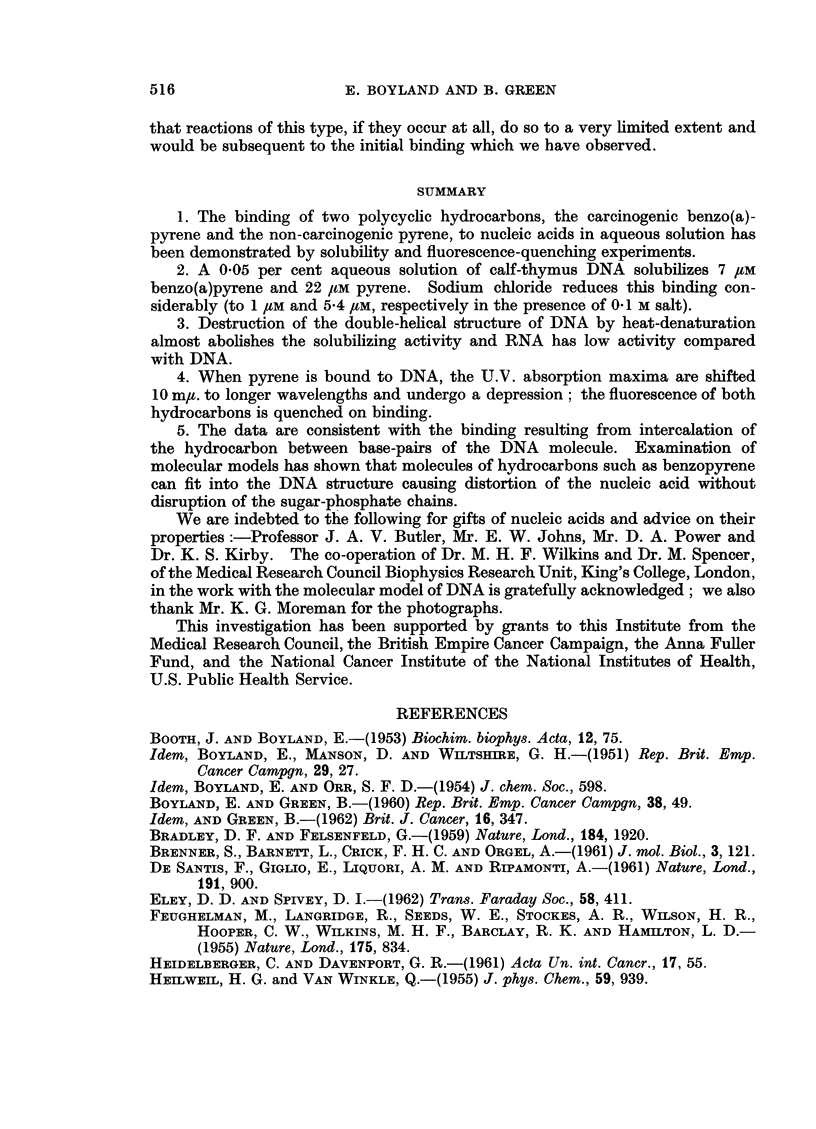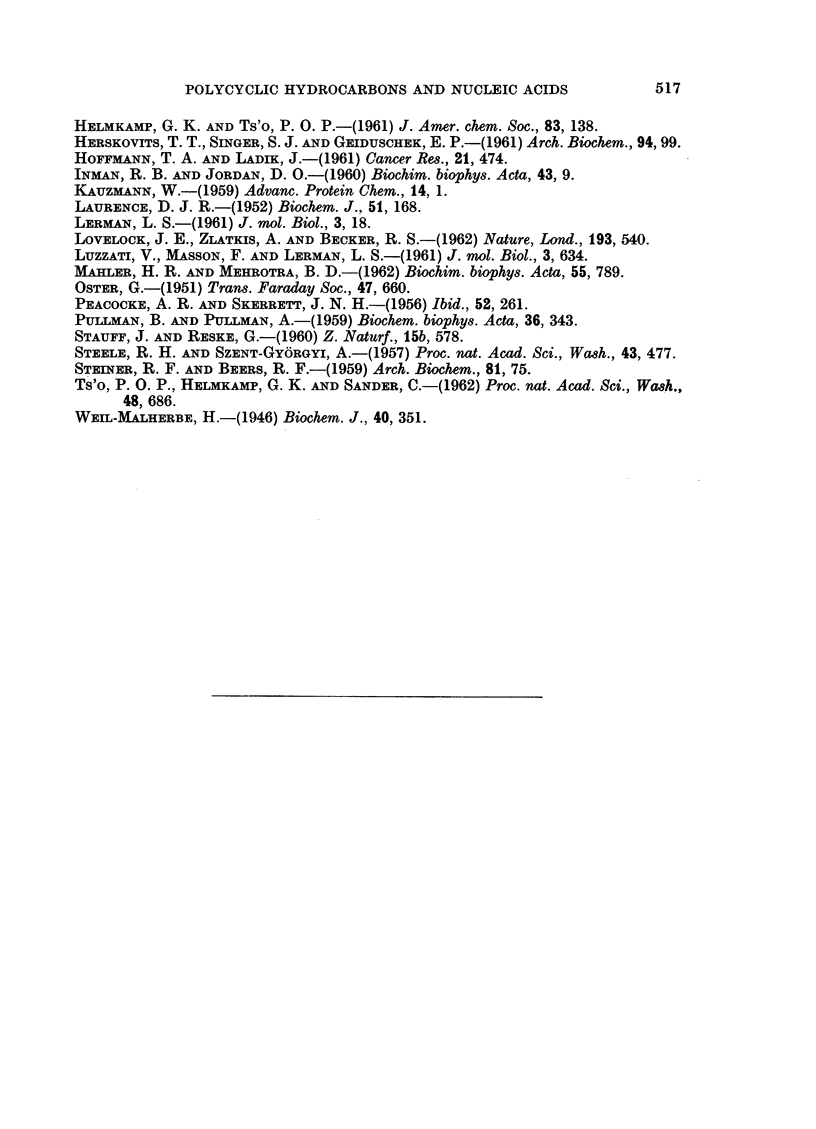# The Interaction of Polycyclic Hydrocarbons and Nucleic Acids

**DOI:** 10.1038/bjc.1962.59

**Published:** 1962-09

**Authors:** E. Boyland, B. Green

## Abstract

**Images:**


### THE INTERACTION OF POLYCYCLIC HYDROCARBONS AND NUCLEIC ACIDS

E. BOYLAND AND B. GREEN

*From the Chester Beatty Research Institute, Institute of Cancer Research : Royal Cancer Hospital, London, S.W.3.*

Received for publication July 4, 1962

THE present study is concerned with the discovery that the carcinogenic hydrocarbon benzo(a)pyrene (3,4-benzopyrene) is more soluble in aqueous DNA solutions than in water (Booth, Boyland, Manson and Wiltshire, 1951). A subsequent quantitative study of the solubility of various nitrogen-containing polycyclic carcinogens (dibenzocarbazoles and dibenzacridines) in aqueous DNA solutions revealed a similar reaction with DNA (Booth and Boyland, 1953). The binding to nucleic acids and polynucleotides of various planar dye molecules [in particular the acridine dyes, acridine orange (e.g. Steiner and Beers, 1959 ; Bradley and Felsenfeld, 1959), acriflavine (Oster, 1951 ; Heilweil and Van Winkle, 1955) and proflavine (Peacocke and Skerrett, 1956 ; Lerman, 1961)] has been investigated in many laboratories and there has been a recent report of the interaction of purine derivatives themselves with nucleic acids (Ts'o, Helmkamp and Sander, 1962).

Almost all the planar molecules for which binding to nucleic acids has been demonstrated, contain hetero-atoms or groups which could provide specific binding sites. The polycyclic hydrocarbons have no such groups, so that the binding of these molecules to nucleic acids is of particular interest quite apart from the obvious implications for the carcinogenic action of certain types. The interaction of the carcinogenic benzo(a)pyrene and non-carcinogenic pyrene with nucleic acids has therefore been investigated in more detail.

### EXPERIMENTAL

### *Materials*

The polycyclic hydrocarbons were those described previously (Boyland and Green, 1962).

The nucleic acids were provided by Professor J. A. V. Butler and Dr. K. S. Kirby. They were in the form of the sodium salts and a 0·05 per cent solution of DNA in water had a sodium ion concentration of about 0·001, which would protect it against spontaneous denaturation. The solutions referred to as 0·05 per cent are based upon the weight of DNA as supplied—without correction for bound water. (The DNA sample used for most experiments had a nitrogen content of 12 per cent and phosphorus content of 6·95 per cent.) Glass-distilled water was used throughout this work.

### *Estimation of solubility of hydrocarbons in nucleic acid solutions*

Excess solid hydrocarbon was shaken with the 0·05 per cent nucleic acid solution for 16 hr. (overnight) at room temperature ($\sim$ 22° C.) in light-shielded flasks.

Initially, hydrocarbon was added to the nucleic acid solution as a small volume of previously-prepared suspension in water (Boyland and Green, 1962). This method gave very low solubilities and it appeared that the amount of hydrocarbon solubilized depended on the particle size, i.e. surface area in contact with the solution (cf. Stauff and Reske, 1960). For later work, excess (*ca.* 5 mg.) solid hydrocarbon was ground with 1–2 ml. of the nucleic acid solution under investigation until the particles were wetted and finely divided and this was then added to the remainder of the solution (10–20 ml.) to be shaken. After shaking, the solution was centrifuged (3,500 r.p.m. for 40 minutes) to remove the suspended solid and the U.V. absorption was measured in the Unicam S.P. 500 spectrophotometer.

Standard solutions were prepared by shaking nucleic acid solutions to which had been added known amounts of hydrocarbon in small volumes of acetone or ethanol, or by depositing a known quantity of hydrocarbon on the base of a flask (by evaporating an ethanol or acetone solution in nitrogen), shaking with nucleic acid solution, decanting off the solution and estimating the quantity of hydrocarbon remaining by dissolving the residue in benzene or *cyclo*hexane and measuring the U.V. absorption of this solution. The concentration of hydrocarbon in the nucleic acid layer was then calculated and the absorption spectrum of this standard solution was measured. A check was provided by taking a portion of the solution in which the hydrocarbon had been estimated, extracting with a known volume of benzene or *cyclo*hexane and measuring the U.V. spectrum of each layer.

In the case of benzopyrene the solubility in water (0·009 $\mu$M) is negligible, whereas that of pyrene (0·8 $\mu$M) is quite considerable. In the standard pyrene solutions, where no large excess of hydrocarbon was present to keep the solution saturated, it was assumed that the proportion of unbound hydrocarbon was not significant. No correction was necessary for the unknown DNA/pyrene solutions since the " bound " pyrene, which was the species estimated, absorbs at 345 m$\mu$. compared with 335 m$\mu$. for pyrene in free aqueous solution. For RNA, where the maxima are depressed but remain at 335 m$\mu$., a correction was applied for the 0·8 $\mu$M pyrene in free solution ; a similar correction was made in the case of denatured DNA.

### *Fluorescence*

All fluorescence measurements were made with the Aminco-Bowman spectrophotofluorimeter.

### RESULTS

The results for the solubilization of the two hydrocarbons by nucleic acid solutions are given in Table I ; the results of the individual determinations are listed to show the variation encountered. In the case of pyrene/DNA in water, the first three solubility estimations gave values of about 8 $\mu$M, whilst the later results consistently gave values of 20–25 $\mu$M. This difference may be due to the use of a pyrene sample with large crystals so that the surface area was proportionately low ; in the first experiments, therefore, it was assumed that these solutions were not saturated and these results were ignored in calculating the mean value. The other results were obtained consistently with at least two different samples of pyrene and nucleic acid.

TABLE I.—*Solubilization of Hydrocarbons by* 0·05 *per cent Nucleic Acid Solutions*

| Nucleic acid | Hydrocarbon Solubilized ($\mu$M): Native ($H_2O$) | Heat-denatured ($H_2O$) | Native (0·1 M NaCl) |
|---|---|---|---|
| (a) *Benzo(a)pyrene* | | | |
| Calf Thymus DNA . . . | 8·5, 4·4, 8·6, 6·6, 4·3, 7·1, 7·1, 8·0 7·1, 5·0. | 0·8, 0·7, 0·5, 0·4 | 0·83, 0·90, 1·2, 0·97 |
| | *Mean 6·7* | *Mean 0·6* | *Mean 0·97* |
| Rat Liver DNA . . . | 3·8 | 0·4 | 0·9 |
| Rat Liver RNA . . . | 0·87, 0·74 | — | — |
| (b) *Pyrene* | | | |
| Calf Thymus DNA . . . | 9·3*, 8·2*, 8·1* 19·8, 20·9, 23·0, 21·9, 24, 25·1, 19·8, 19·4 | 0·4, 0·4, 0·5, 0·2 | 7·5, 5·7, 4·2, 5·0 4·3 |
| | *Mean 21·7* | *Mean 0·4* | *Mean 5·4* |
| Rat Liver RNA . . . | 1·2, 1·6, 1·6 | | 0·9 |

* Omitted in calculating mean value—see text.

There is considerable solubilization of both hydrocarbons by DNA in aqueous solution. Taking the DNA solution as 0·14 $\mu$M, this represents roughly 50 benzopyrene molecules and 150 pyrene molecules bound per DNA molecule—and, taking roughly 4,500 base-pairs per DNA molecule, is equivalent to one benzopyrene molecule per 90 base-pairs or one per 30 base-pairs for pyrene. It is possible the amount of hydrocarbon bound could be increased by lowering the sodium concentration still further but this may result in spontaneous denaturation of the nucleic acid.

Increasing the sodium ion concentration reduces in dramatic fashion the amount of hydrocarbon binding to DNA. For 0·1 M sodium chloride the reduction is seven-fold for benzopyrene and rather less (four-fold) for pyrene ; for 0·01 M sodium chloride the reductions are almost as great (to 1·4 and 7 $\mu$M respectively). This is not a simple solubility effect since the solubility of benzopyrene in water is not greatly affected by these concentrations of salt.

Destruction of the rigid double-helical structure of DNA by heat-denaturation virtually abolishes the solubilization effect and, as one might expect, RNA, which has a low helical content, has very little activity. (The figures for these latter two, where the solubilities are so low, should be taken as maximum values.) The forces involved in the binding are relatively weak, since both hydrocarbons can be removed by extracting the aqueous DNA solution with *cyclo*hexane.

*Spectral changes.*—The actual U.V. absorption spectra obtained in one experiment are shown in Fig. 1 and 2. When pyrene is bound to DNA, the U.V. absorption spectrum is modified. The maxima are shifted by 10 m$\mu$., towards longer wavelengths and depressed so that the extinction is reduced to half that of free pyrene. Fig. 3 shows the effect on the spectrum of pyrene in water of 0·06 M (1·17 per cent) caffeine and 0·05 per cent DNA. Thus DNA causes changes in the hydrocarbon spectra similar to those induced by purines (cf. Booth and Boyland, 1953 ; Boyland and Green, 1962), but they are more pronounced. RNA produces a depression in the pyrene maxima of 33 per cent but no shift to longer wavelengths and heat-denatured DNA also produces no bathochromic shift.

The spectral changes induced by DNA were not produced (at comparable concentrations) in the non-aqueous solvents ethylene glycol, and formamide, where the double-helical structure of DNA is abolished (Helmkamp and Ts'o, 1961).

The solubility of benzopyrene in water is too small for direct spectral measurement but the spectra in nucleic acid solutions appear to follow a similar pattern

FIG. 1.—U.V. absorption spectra of aqueous 0·05 per cent nucleic acid solutions shaken with excess solid pyrene and centrifuged. Curve 1—Native DNA in $H_2O$. Curve 2—Native DNA in 0·1 M NaCl. Curve 3—Heat-denatured DNA in water. Upper Curve RNA.

to those of pyrene (Fig. 2). The maxima in DNA are at 375 mμ. and 395 mμ. and in 0·06 M caffeine at 371 mμ and 391 mμ.

*Fluorescence quenching*

Nucleic acids quench the fluorescence of both pyrene and benzopyrene in aqueous solution, as can be seen in Fig. 4 and 5. The quenching action is again most efficient in water but is greatly reduced in the presence of 0·1 M sodium chloride or if RNA replaces DNA as quenching agent.

Because of the low solubility of the hydrocarbons in water, the quenching studies have had to be made on particularly dilute solutions. The benzopyrene curves, for example, were obtained at hydrocarbon concentrations of 5–6 $\mu$g./litre ; even this is higher than the actual solubility previously determined (Boyland and Green, 1962) but it was assumed that little precipitation would occur at these low concentrations. Pyrene was investigated at concentrations of 40 or

FIG. 2.—U.V. absorption spectra of aqueous 0·05 per cent nucleic acid solutions shaken with excess solid benzo(a)pyrene and centrifuged. Curve 1—Native DNA in $H_2O$. Curve 2—Native DNA in 0·1 M NaCl. Curve 3—Heat-denatured DNA in water. Upper curve—RNA.

80 $\mu$g./litre. Care must be taken in interpreting the curves obtained because any complex could be dissociated by dilution alone at these concentrations—this is also true for the ionic complexes of acridines and DNA (Bradley and Felsenfeld, 1959).

The DNA curves represent the equilibria between hydrocarbon in free solution and that bound to DNA, since it is clear from the solubility figures that there are unoccupied binding sites at DNA concentrations where the hydrocarbon fluorescence is not fully quenched.

Another problem is that at the lower DNA concentrations in water there may be some denaturation due to dilution (Inman and Jordan, 1960); the initial curvature in the pyrene quenching curve (i.e. at very low DNA concentrations) may be the result of this effect. In this solution the fluorescence quenching is proportional to DNA concentration over the major part of the curves, increasing rapidly to levels where the fluorescence is too close to the background fluorescence for $F_0/F$ to be measured accurately, especially for benzopyrene. RNA is

FIG. 3.—The effect of caffeine (1·17 per cent–0·06 M) and DNA (0·05 per cent) on the absorption spectrum of pyrene (0·8 μM) in water.

FIG. 4.—The quenching of pyrene fluorescence by nucleic acids in aqueous solution. Pyrene concentration 40 μg./litre. $F_0$ = Fluorescence intensity in absence of quencher. F = Fluorescence intensity in presence of stated concentration of nucleic acid.

also moderately effective as a quenching agent, the quenching curve rising less steeply than for DNA and tending to plateau above certain concentrations where maximum binding is attained.

For benzopyrene the large differences in $F_0/F^{-1}$ levels for RNA and DNA in water and salt result from comparatively small absolute differences in the values of F for the fully quenched solutions, where the fluorescence intensities are so low.

At present insufficient information is available on these dilute systems for a detailed analysis of the curves to be possible but since the order of quenching

FIG. 5.—The quenching of benzo(a)pyrene fluorescence by nucleic acids in aqueous solution. $F_0$ = Fluorescence intensity in absence of quencher. F = Fluorescence intensity in presence of stated concentration of nucleic acid.

efficiencies is the same as that of solubilizing efficiencies for each hydrocarbon the same process (binding to nucleic acids) may be assumed to cause both phenomena.

Quenching of the hydrocarbon fluorescence by DNA is not observed at similar concentrations in methanol or formamide where the nucleic acid has lost its secondary structure (Herskovits, Singer and Geiduschek, 1961 ; Helmkamp and Ts'o, 1961).

### DISCUSSION

The finding that appreciable amounts of polycyclic hydrocarbons bind to DNA in aqueous solutions is perhaps unexpected in view of previous reports of slight or indefinite activity (Booth *et al.*, 1951 ; Steele and Szent-Györgyi, 1957 ; Stauff and Reske, 1960). In fact about 50 benzopyrene or 150 pyrene molecules are bound per DNA molecule and it is possible that still more hydrocarbon would go into solution if the conditions were suitably adjusted. Pyrene, being more water-soluble than the larger benzopyrene, might be expected to go into solution with DNA more readily but the contrast between the 80-fold difference in water-solubility of the hydrocarbons and the 3-fold difference in binding to DNA, leads to the speculation that the benzopyrene molecule is more firmly bound. One would expect that the binding of such large, planar, non-polar molecules with no obvious single mode of binding to individual groups would involve the relatively hydrophobic " core " of stacked bases of the DNA so that the greatest stabilization would be gained from the hydrophobic effect ; (for discussions of this type of binding, see e.g. Laurence, 1952 ; Kauzmann, 1959).

Three types of binding mechanism appear feasible. These are (1) non-specific " adsorption " to the external surface of the stacked bases (Feughelman, Langridge, Seeds, Stockes, Wilson, Hooper, Wilkins, Barclay and Hamilton, 1955); (2) interaction with planar purine bases in regions where they are not involved in the double-helix (i.e. a similar interaction to that observed in simple aqueous purine solutions by Weil-Malherbe, 1946, and Boyland and Green, 1962); (3) intercalation between base-pairs.

Reaction (2) is ruled out as a major contributor to DNA solubilization by the low activity of RNA or heat-denatured DNA but it could account for the solubilizing activity of these two, where there is no shift in the hydrocarbon spectra. The choice is then between (1) and (3). Through the co-operation of Dr. M. H. F. Wilkins and Dr. M. Spencer of King's College, London, we were able to show (Boyland and Green, 1960) that planar polycyclic hydrocarbon molecules such as benzopyrene or dibenzanthracene could be accommodated between base-pairs of DNA without disrupting the sugar-phosphate side-chains (Fig. 6*a* and *b*). The side-view (Fig. 6*b*) shows that, as a result of " untwisting " of the bases, the side-chains, instead of spiralling normally, are " straightened " over the region where the hydrocarbon is inserted; the normal structure is then resumed once more. The interactions would be similar to those previously observed for individual purines and hydrocarbons in aqueous solution—polarization forces stabilized by an overall hydrophobic effect.

This mode of binding has been examined in more rigorous fashion by Lerman (Lerman, 1961; Luzzati, Masson and Lerman, 1961) for the interaction of DNA and acridine derivatives and, in the case of proflavin, direct experimental evidence in favour of intercalation has been obtained. Although in the present work direct proof for this mode of binding of hydrocarbons has not been obtained, the fact that the spectral changes are similar to, but more pronounced than, those induced by individual purines in aqueous solution [where there is almost certainly a loose " sandwich " arrangement of planar purine and hydrocarbon molecules (Booth, Boyland and Orr, 1954; Boyland and Green, 1962; De Santis, Giglio, Liquori and Ripamonti, 1961)], together with the requirement for the intact double-helical structure, favour intercalation.

Sodium chloride would tend to suppress the phosphate-group repulsions and possibly, as a result of rendering non-polar substances less soluble, strengthen the hydrophobic bonding between the stacked bases now considered important for the stability of the DNA secondary structure (Herskovits, *et al.*, 1961; Mahler and Mehrotra, 1962). This increased stability would render sites in the hydrophobic core less accessible to the hydrocarbon. It is interesting that interaction of purines and pyrimidines themselves with DNA was not apparently by intercalation (Ts'o, *et al.*, 1962).

The binding of radioactivity to DNA fractions of mouse-skin following skin-painting with labelled 1,2 : 5,6-dibenzanthracene has been demonstrated by

---

EXPLANATION OF PLATES

FIG. 6.—Photographs of molecular models showing how a benzopyrene molecule (in yellow) can be inserted between the base-pairs of DNA without rupturing the sugar-phosphate chains. The DNA model, from King's College, London, was used by courtesy of Dr. Wilkins.

6b

6a

Boyland and Green.

Heidelberger and Davenport (1961). This binding was equivalent to only 1·5 hydrocarbon molecules per DNA molecule. The radioactivity could be released after enzymatic digestion, to which, however, the DNA was rendered more resistant. This binding was quite firm, since the extensive washings with organic solvents employed in the preparation of the fractions would release hydrocarbon bound in a manner similar to that found in our *in vitro* experiments, unless it were protected by the protein which was present during these washings before final purification. Recent work with tritiated benzopyrene by Brookes and Lawley (personal communication) has indicated a similar firm binding of activity to mouse-skin DNA fractions which contained little, if any, protein. The relation of this binding, which apparently involves some metabolic process, to that reported here is not clear at present.

Although in general, in work with carcinogens, stress has been laid on " firm chemical " binding to tissue constituents it is clear that " loose " complexing of the type described in this paper could have far-reaching effects in a living cell. Conditions in the cell must differ from the simple systems with which the present paper is concerned ; the effect on complexing of the protein which invariably accompanies DNA in the cell is unknown, but since such loose binding by its very nature would be difficult to study in tissues, it is reasonable to consider the possible consequences, assuming it to occur as it does in the test-tube.

Following the work of Lerman on acridine derivatives, Brenner, Barnett, Crick and Orgel (1961) suggested that if an acridine molecule were intercalated between the bases of one pair and not the other during replication, this could lead to addition or subtraction of a base and one could predict deletion or addition of a base-pair. A similar argument could be applied to the polycyclic hydrocarbons, but this simple picture obviously could not explain the carcinogenic action of hydrocarbons since the non-carcinogenic pyrene is bound in an apparently similar manner to the carcinogenic benzopyrene. For this type of explanation to be tenable additional postulates would be needed. These could include, (1) only benzopyrene is attached to the site which is susceptible to carcinogenic change in a particular tissue, (2) the manner of binding at a given site is different—perhaps carcinogenic hydrocarbons are more firmly bound, (3) pyrene is less available for DNA-binding in the cell because of a deficient transport mechanism or because it is metabolised too rapidly.

Another possibility which has excited some theoretical interest is in the type of interaction which could occur between the base-pairs and intercalated hydrocarbon and in particular the possibility of charge-transfer interactions (Pullman and Pullman, 1959 ; Hoffmann and Ladik, 1961). The hydrocarbon is usually suggested as the acceptor molecule and the work of Lovelock, Zlatkis and Becker (1962) has demonstrated the affinity of polycyclic hydrocarbons for thermally excited electrons. The effects of such an interaction could be transferred at least along certain localized regions of the helix as a result of the electronic structure of the stacked bases of the DNA molecule (Eley and Spivey, 1962) perhaps resulting in a decreased stability of the molecule.

We have found no evidence to suggest that charge-transfer is important in the solubilization of benzopyrene or pyrene by DNA. No definite spectral bands corresponding to such transfer have been observed (although it ought to be pointed out that they are not invariably observed in charge-transfer reactions and the solutions with which we are dealing are very dilute). It is reasonable to assume that reactions of this type, if they occur at all, do so to a very limited extent and would be subsequent to the initial binding which we have observed.

SUMMARY

1. The binding of two polycyclic hydrocarbons, the carcinogenic benzo(a)-pyrene and the non-carcinogenic pyrene, to nucleic acids in aqueous solution has been demonstrated by solubility and fluorescence-quenching experiments.

2. A 0·05 per cent aqueous solution of calf-thymus DNA solubilizes 7 $\mu$M benzo(a)pyrene and 22 $\mu$M pyrene. Sodium chloride reduces this binding considerably (to 1 $\mu$M and 5·4 $\mu$M, respectively in the presence of 0·1 M salt).

3. Destruction of the double-helical structure of DNA by heat-denaturation almost abolishes the solubilizing activity and RNA has low activity compared with DNA.

4. When pyrene is bound to DNA, the U.V. absorption maxima are shifted 10 m$\mu$. to longer wavelengths and undergo a depression ; the fluorescence of both hydrocarbons is quenched on binding.

5. The data are consistent with the binding resulting from intercalation of the hydrocarbon between base-pairs of the DNA molecule. Examination of molecular models has shown that molecules of hydrocarbons such as benzopyrene can fit into the DNA structure causing distortion of the nucleic acid without disruption of the sugar-phosphate chains.

We are indebted to the following for gifts of nucleic acids and advice on their properties :—Professor J. A. V. Butler, Mr. E. W. Johns, Mr. D. A. Power and Dr. K. S. Kirby. The co-operation of Dr. M. H. F. Wilkins and Dr. M. Spencer, of the Medical Research Council Biophysics Research Unit, King's College, London, in the work with the molecular model of DNA is gratefully acknowledged ; we also thank Mr. K. G. Moreman for the photographs.

This investigation has been supported by grants to this Institute from the Medical Research Council, the British Empire Cancer Campaign, the Anna Fuller Fund, and the National Cancer Institute of the National Institutes of Health, U.S. Public Health Service.

### REFERENCES

Booth, J. and Boyland, E.—(1953) *Biochim. biophys. Acta*, **12**, 75.

*Idem*, Boyland, E., Manson, D. and Wiltshire, G. H.—(1951) *Rep. Brit. Emp. Cancer Campgn*, **29**, 27.

*Idem*, Boyland, E. and Orr, S. F. D.—(1954) *J. chem. Soc.*, 598.

Boyland, E. and Green, B.—(1960) *Rep. Brit. Emp. Cancer Campgn*, **38**, 49.

*Idem*, and Green, B.—(1962) *Brit. J. Cancer*, **16**, 347.

Bradley, D. F. and Felsenfeld, G.—(1959) *Nature, Lond.*, **184**, 1920.

Brenner, S., Barnett, L., Crick, F. H. C. and Orgel, A.—(1961) *J. mol. Biol.*, **3**, 121.

De Santis, F., Giglio, E., Liquori, A. M. and Ripamonti, A.—(1961) *Nature, Lond.*, **191**, 900.

Eley, D. D. and Spivey, D. I.—(1962) *Trans. Faraday Soc.*, **58**, 411.

Feughelman, M., Langridge, R., Seeds, W. E., Stockes, A. R., Wilson, H. R., Hooper, C. W., Wilkins, M. H. F., Barclay, R. K. and Hamilton, L. D.—(1955) *Nature, Lond.*, **175**, 834.

Heidelberger, C. and Davenport, G. R.—(1961) *Acta Un. int. Cancr.*, **17**, 55.

Heilweil, H. G. and Van Winkle, Q.—(1955) *J. phys. Chem.*, **59**, 939.

HELMKAMP, G. K. AND TS'O, P. O. P.—(1961) *J. Amer. chem. Soc.*, **83**, 138.
HERSKOVITS, T. T., SINGER, S. J. AND GEIDUSCHEK, E. P.—(1961) *Arch. Biochem.*, **94**, 99.
HOFFMANN, T. A. AND LADIK, J.—(1961) *Cancer Res.*, **21**, 474.
INMAN, R. B. AND JORDAN, D. O.—(1960) *Biochim. biophys. Acta*, **43**, 9.
KAUZMANN, W.—(1959) *Advanc. Protein Chem.*, **14**, 1.
LAURENCE, D. J. R.—(1952) *Biochem. J.*, **51**, 168.
LERMAN, L. S.—(1961) *J. mol. Biol.*, **3**, 18.
LOVELOCK, J. E., ZLATKIS, A. AND BECKER, R. S.—(1962) *Nature, Lond.*, **193**, 540.
LUZZATI, V., MASSON, F. AND LERMAN, L. S.—(1961) *J. mol. Biol.*, **3**, 634.
MAHLER, H. R. AND MEHROTRA, B. D.—(1962) *Biochim. biophys. Acta*, **55**, 789.
OSTER, G.—(1951) *Trans. Faraday Soc.*, **47**, 660.
PEACOCKE, A. R. AND SKERRETT, J. N. H.—(1956) *Ibid.*, **52**, 261.
PULLMAN, B. AND PULLMAN, A.—(1959) *Biochem. biophys. Acta*, **36**, 343.
STAUFF, J. AND RESKE, G.—(1960) *Z. Naturf.*, **15*b***, 578.
STEELE, R. H. AND SZENT-GYÖRGYI, A.—(1957) *Proc. nat. Acad. Sci., Wash.*, **43**, 477.
STEINER, R. F. AND BEERS, R. F.—(1959) *Arch. Biochem.*, **81**, 75.
TS'O, P. O. P., HELMKAMP, G. K. AND SANDER, C.—(1962) *Proc. nat. Acad. Sci., Wash.*, **48**, 686.
WEIL-MALHERBE, H.—(1946) *Biochem. J.*, **40**, 351.